# Dual-antigen recognition iPSC-derived CAR-T cells for B-cell malignancies: establishment of a COVID-19 vaccine synergy strategy

**DOI:** 10.3389/fcell.2026.1772146

**Published:** 2026-02-26

**Authors:** Norihide Izumi, Yoshiki Furukawa, Shintaro Kinoshita, Midori Ishii, Ayaka Goto, Hajime Yasuda, Jun Ando, Hiromitsu Nakauchi, Miki Ando

**Affiliations:** 1 Department of Hematology, Juntendo University School of Medicine, Tokyo, Japan; 2 Division of Cell Therapy and Blood Transfusion Medicine, Juntendo University School of Medicine, Tokyo, Japan; 3 Stem Cell Therapy Division, Advanced Research Initiative, Institute of Science Tokyo, Tokyo, Japan; 4 Institute of Stem Cell Biology and Regenerative Medicine, Stanford University School of Medicine, Stanford, CA, United States

**Keywords:** CAR-T, iPSC-derived T-cells, SARS-CoV-2, TCR restimulation, vaccine synergy

## Abstract

**Introduction:**

CD19-directed chimeric antigen receptor (CD19-CAR) T-cell therapy has markedly improved outcomes in relapsed and refractory B-cell malignancies, but its efficacy remains limited by insufficient *in vivo* persistence and functional exhaustion. We have generated functionally rejuvenated T-cells (rejTs) by reprogramming antigen-specific cytotoxic T lymphocytes (CTLs) into induced pluripotent stem cells (iPSCs) and redifferentiating them into CTLs with restored proliferative capacity. In this study, we explored a vaccine synergy strategy to enhance the persistence of CAR-rejuvenated CTLs (CARrejTs) through T-cell receptor (TCR) restimulation.

**Methods:**

SARS-CoV-2 spike protein-specific rejTs (COVID19-rejTs) were established from iPSCs derived from spike protein-specific CTLs. A CD19-CAR was introduced into these iPSCs to generate dual-antigen recognition CARrejTs targeting CD19 and COVID-19 spike protein (1919-CARrejTs). Subsequently, 1919-CARrejTs were assessed for cytotoxicity, proliferative capacity, and exhaustion phenotype using ^51^Cr release assays, sequential rechallenge assays, and CFSE-based proliferation analysis with CAR- or TCR-dependent stimulation.

**Results:**

1919-CARrejTs uniformly expressed both CD19-CAR and spike protein-specific TCRs, retained antigen-specific cytotoxicity, and exhibited a rejuvenated phenotype with higher expression of granzyme B and perforin and lower expression of exhaustion markers compared with conventional CD19-CAR-T cells. Dual-antigen recognition enhanced cytotoxicity under matched antigen presentation, and 1919-CARrejTs maintained durable tumor control in sequential rechallenge assays. CFSE dilution analysis revealed that TCR-mediated stimulation by spike protein-specific peptide provided strong proliferative capacity of 1919-CARrejTs in an HLA-dependent manner.

**Conclusion:**

The combination of iPSC-mediated rejuvenation and dual-antigen recognition via CAR and native TCR confers superior cytotoxicity, persistence, and proliferative potential compared to conventional CD19-CAR-T cells. These findings provide a proof-of-concept for a vaccine-synergy strategy in which *in vivo* TCR restimulation supports selective expansion and sustained antitumor effect of dual-antigen recognition T-cells that can be a promising treatment approach for B-cell malignancies.

## 1 Introduction

Chimeric antigen receptor (CAR) T-cell therapy has markedly improved remission rates and prolonged survival in relapsed and refractory B-cell malignancies (Cappell and Kochenderfer, 2023; Neelapu et al., 2023). Although durable remissions can be achieved in around 50% of these patients, insufficient *in vivo* persistence and CAR-T cell exhaustion remain as obstacles (Cappell and Kochenderfer, 2023; Kouro et al., 2022). In recent years, the concept of “vaccine synergy” has been introduced as a strategy to address these limitations. Enhancing CAR-T cell expansion, persistence, and function *in vivo* by antigen restimulation through vaccinations has gained increasing attention (Li et al., 2025). Utilization of multiple vaccine platforms including mRNA, peptides, viral vectors, and dendritic cells (DC) have been reported and early-phase clinical trials show improvements in CAR-T cell re-expansion and disease control through mechanisms such as T-cell activation, induction of memory-like phenotypes, and suppression of immune evasion (Caruana et al., 2015; Evgin et al., 2022; Li et al., 2025; Sun et al., 2023). SARS-CoV-2 mRNA vaccines are globally used and administered periodically, making them an extremely accessible and safe source of T-cell receptor (TCR) stimulation (Berthaud et al., 2024; Polack et al., 2020). Spike protein-specific CD8^+^ T cells have been reported to increase in the peripheral blood of healthy individuals after vaccination, indicating that antigen-specific TCR engagement can induce expansion of the corresponding cytotoxic T lymphocytes (CTL) fraction (Aoki et al., 2024; Oberhardt et al., 2021). This observation suggests that TCR stimulation can support proliferation and persistence of CAR-T cells *in vivo* (Wachsmann et al., 2022).

We previously demonstrated that by utilizing induced pluripotent stem cell (iPSC) technology, antigen specific CTLs can be functionally rejuvenated (rejuvenated CTLs; rejTs), and these cells exhibit long-term persistence *in vivo* due to reduced T-cell exhaustion and acquisition of a younger memory-like phenotype compared to the original CTLs (Ando et al., 2020; Ando et al., 2015; Nishimura et al., 2013). Next, we introduced CD19-CAR into iPSC-derived Epstein-Barr virus (EBV) latent membrane protein (LMP)-2 specific T-cells and generated CAR-rejuvenated CTLs (CARrejTs) that can target dual-antigens via native TCR and CAR (Harada et al., 2022). These CARrejTs not only express the CD19-CAR but also harbor an LMP2-specific TCR, enabling them to proliferate and undergo memory differentiation in response to EBV exposure or vaccination, thereby providing the potential for sustained and effective antitumor activity (Harada et al., 2022). Importantly, iPSCs possess an essentially unlimited proliferative capacity, allowing them to serve as a sustainable source for CAR-T cell generation and enables the development of “off-the-shelf” CAR-T therapies (Ando and Nakauchi, 2017). Coronavirus disease 2019 (COVID-19), which is caused by the severe acute respiratory syndrome coronavirus 2 (SARS-CoV-2) (Wiersinga et al., 2020). COVID-19 continues to persist as a global infectious disease, and we hypothesized that combining a vaccine synergy strategy with CARrejTs retaining native TCR specificity targeting the SARS-CoV-2 spike protein could enhance their *in vivo* persistence and proliferation through TCR restimulation. Therefore, we introduced CD19-CAR into iPSC-derived SARS-CoV-2 spike protein specific CTLs (COVID19-rejTs) and generated dual-antigen recognition CARrejTs targeting CD19 and COVID-19 spike protein (1919-CARrejTs) and evaluated the cytotoxicity, durability of tumor suppression, and proliferative capacity induced by TCR and CAR stimulation.

## 2 Materials and methods

### 2.1 Human samples and SARS-CoV-2 vaccination

This study was conducted in accordance with the Declaration of Helsinki and was approved by the Ethics Committee at Juntendo University School of Medicine. Peripheral blood samples were obtained from healthy adult volunteers carrying HLA-A*02:01 or HLA-A*24:02, and donors received the BNT162b2 SARS-CoV-2 mRNA vaccine (BioNTech/Pfizer, Mainz, Germany/New York, NY) according to standard protocols. Peripheral blood mononuclear cells (PBMCs) were collected from unvaccinated individuals (defined as more than 1 year after their last vaccination) and vaccinated individuals (defined as vaccinations within 3 months after vaccination).

### 2.2 Cell lines

The acute lymphoid leukemia cell line NALM-6 (RRID: CVCL_0092), Burkitt lymphoma cell line Raji (RRID: CVCL_0511), and pancreatic cancer cell line PANC-1 (RRID: CVCL_0480) were purchased from RIKEN BioResource Center (Tsukuba, Japan). The non-small cell lung cancer cell line A549 (RRID: CVCL_0023) was purchased from ATCC (Manassas, VA). Lymphoblastoid cell lines (LCLs) were derived from PBMCs in our laboratory using EBV isolated from B95-8 cell lines in the presence of cyclosporin A. NALM-6, Raji, PANC-1, and LCLs were cultured under standard conditions at 37 °C and 5% CO_2_ using Roswell Park Memorial Institute 1640 medium (Gibco, Carlsbad, CA) supplemented with 10% FBS (Gibco), 100 U/mL penicillin, 100 μg/mL streptomycin, and 2 mM L-glutamine (Gibco). A549 cells were cultured under standard conditions in Dulbecco’s modified Eagle medium (DMEM; Gibco) containing 10% FBS and antibiotics as described above.

### 2.3 Vector construction and virus production

To generate a lentiviral Cs-Ubc-CD19-4-1BB-CD3ζ-F2A-mCherry construct, CD19-4-1BB-CD3ζ was amplified by PCR from the retroviral MSCV-CD19-4-1BB-CD3ζ-IRES-GFP plasmid (Harada et al., 2022) and fused with a lentiviral Cs-Ubc-iC9-F2A-mCherry construct (Ando et al., 2015) by replacing iC9 with CD19-4-1BB-CD3ζ using InFusion cloning (Takara Bio) (Figure 3A). After Cs-Ubc-CD19-4-1BB-CD3ζ-F2A-mCherry was generated, 293T cells were transfected with the lentiviral construct together with the packaging plasmids pMDLg/pRRE and pCMV-VSVG-RSV-Rev, using polyethylenimine (Polysciences, Warrington, PA) as the transfection reagent. Virus supernatants were collected 48–72 h after application of the forskolin-containing medium (Ando et al., 2014).

### 2.4 Peripheral blood-derived CAR-T generation

PBMCs obtained from a healthy donor harboring HLA-A*02:01 were activated with T Cell TransAct (Miltenyi Biotec, Bergisch Gladbach, Germany) in the presence of recombinant human IL-2 (100 U/mL). After 3 days, activated T-cells were transferred onto 24-well plates coated with RetroNectin (Takara Bio, Shiga, Japan) and CD19-CAR lentiviral supernatants were added to each well. CAR-T cells were cultured in NS-A2 medium (Nissui, Tokyo, Japan) supplemented with 10 ng/mL each of IL-7 (Miltenyi Biotec) and IL-15 (Miltenyi Biotec).

### 2.5 Generation of SARS-CoV-2 spike protein-specific CTLs and establishment of iPSCs

PBMCs from an HLA-A*02:01-positive healthy donor were cocultured with autologous DCs pulsed with the SARS-CoV-2 spike protein_269-277_ peptide (YLQPRTFLL; S269), whereas PBMCs from an HLA-A*24:02-positive donor were cocultured with DCs pulsed with the spike protein_448-456_ peptide (NYNYLYRLF; S448) (Mimotopes, Victoria, Australia) in the presence of IL-4 (400 IU/mL) and IL-7 (10 ng/mL) as described previously (Gerdemann et al., 2012; Rooney et al., 2014). On day 9 of culture, T-cells were restimulated with peptide-pulsed DCs. On day 16, CTLs were stained with HLA-A*02:01/S269 tetramer or HLA-A*24:02/S448 tetramer (MBL, Nagoya, Japan). Tetramer-positive CTLs were cloned by a limiting dilution to obtain SARS-CoV-2-specific CTL clones (Ando et al., 2020; Ando et al., 2015; Nishimura et al., 2013).

### 2.6 Establishment of T-iPSCs from SARS-CoV-2-specific CTLs

A SARS-CoV-2-specific CTL clone recognizing the HLA-A*02:01-restricted S269 (COVID19-CTL) was reprogrammed using Sendai virus vectors encoding OCT3/4, SOX2, KLF4, c-MYC, NANOG, and LIN28 (Honda et al., 2020). Transduced cells were transferred onto plates coated with iMatrix-511 (Nippi, Tokyo, Japan) and cultured in NS-A2 medium. Growth medium was gradually replaced with StemFit AK03N (Ajinomoto Healthy Supply, Tokyo, Japan). After 14 days, emerging iPSC colonies were picked up into new culture wells and passaged in CTS Essential 8 medium (Thermo Fisher Scientific, Waltham, MA, United States) several times until cell numbers sufficed for downstream experiments (Ando et al., 2020). The established T-iPSC line derived from COVID19-CTL was designated as COVID19-iPSCs.

### 2.7 CD19-CAR transduction into T-iPSCs

COVID19-iPSCs (1 × 10^5^ cells) were transduced with CD19-CAR lentiviral vector on vitronectin (Thermo Fisher Scientific)-coated plates (multiplicity of infection of 20) and cultured in CTS Essential 8 medium (Thermo Fisher Scientific). On day 7, mCherry + iPSCs were isolated using a MoFlo Astrios EQ cell sorter (Beckman Coulter, Brea, CA). The CD19-CAR^+^ iPSC (CD19CAR-COVID19-iPSCs) population was subsequently enriched by repeated sorting to increase CAR positivity.

### 2.8 Differentiation of T-iPSCs into rejTs

Two types of rejTs were generated, 1919-CARrejTs derived from CD19-CAR-COVID19-iPSCs, and COVID19-rejTs derived from COVID19-iPSCs. Small clumps of T-iPSCs were plated on C3H10T1/2 feeder cells in IMDM supplemented with gamma-irradiated 15% FBS (HyClone, GE Healthcare UK, Little Chalfont, UK) and a cocktail of 10 mg/mL insulin, 5.5 mg/mL transferrin, 5 ng/mL sodium selenite, 2 mM L-glutamine (Thermo Fisher Scientific), 0.45 mM α-monothioglycerol (Sigma-Aldrich, St. Louis, MO), and 50 mg/mL ascorbic acid (Takeda Pharmaceutical, Tokyo, Japan) in the presence of 20 ng/mL vascular endothelial growth factor (VEGF; Miltenyi Biotec). On day 14 of coculture, sac-like structures that contained hematopoietic progenitor cells were extracted and transferred onto C3H10T1/2 feeder cells expressing Delta-like ligand 1 (DLL1) and DLL4 in minimum essential medium (MEM) α (Thermo Fisher Scientific) with FBS (HyClone) in the presence of 20 ng/mL human stem cell factor, 10 ng/mL human Fms-related tyrosine kinase 3 ligand (both Miltenyi Biotec), and 10 ng/mL IL-7. On day 28 of coculture, T-lineage cells were harvested, stimulated by irradiated PBMCs in NS-A2 in the presence of 5 mg/mL phytohemagglutinin (Sigma-Aldrich) and of IL-7 and IL-15 (10 ng/mL each). SARS-CoV-2 specificity and CD19-CAR transgene expression were confirmed by staining with an HLA-A*02:01/S269 tetramer and by detecting mCherry fluorescence, respectively.

### 2.9 ^51^Cr release assays

**FIGURE 1:**
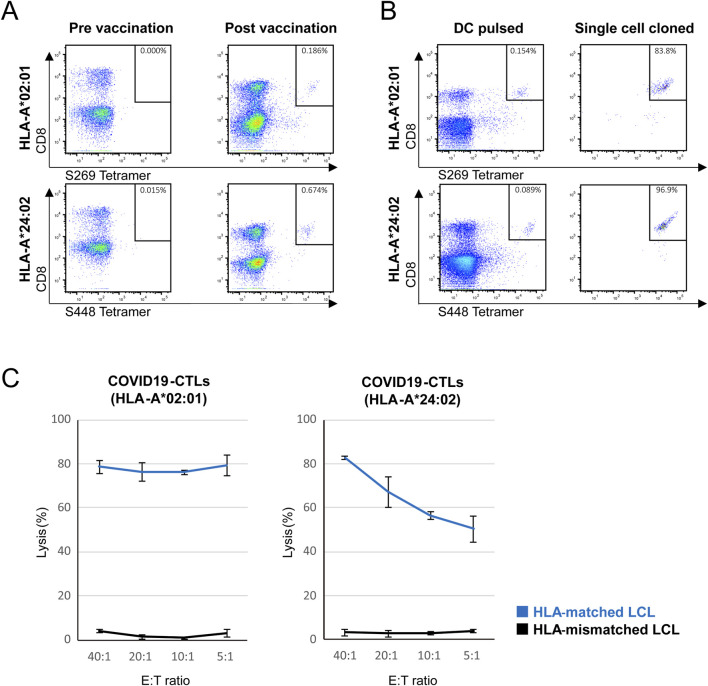
SARS-CoV 2 mRNA vaccination induces spike protein-specific CD8^+^ T cells in healthy donors and allows *in vitro* generation of functional spike-protein specific CTL clones. **(A)** Flow cytometric analysis of PBMCs from HLA-A*02:01 and HLA-A*24:02 donors before and after SARS-CoV-2 mRNA vaccination, to detect peptide-specific CD8^+^ T cells using PE-conjugated HLA-A*02:01/S269 and HLA-A*24:02/S448 tetramers. PBMCs were obtained from one HLA-A*02:01 donor and one HLA-A*24:02 donor. **(B)** Flow cytometric analysis of PBMCs from HLA-A*02:01 and HLA-A*24:02 donors cocultured with autologous dendritic cells pulsed with the corresponding SARS-CoV-2 spike peptides (S269 for A*02:01; S448 for A*24:02), to detect peptide-specific CD8^+^ T cells using PE-conjugated HLA-A*02:01/S269 or HLA-A*24:02/S448 tetramers. Left panels: after peptide-pulsed DC coculture. Right panels: following single-cell cloning of tetramer-reactive CTLs. PBMCs were obtained from one HLA-A*02:01 donor and one HLA-A*24:02 donor. Pre- and post-vaccination samples were paired within the same donor for each HLA type. Data are representative of three independent experiments. **(C)** *In vitro* ^51^Cr-release assay of COVID19-CTLs from HLA-A*02:01 (left) and HLA-A*24:02 (right) donors. CTLs were tested against HLA-matched (blue) or -mismatched (black) LCL targets pulsed with the S269 or S448 peptide. Target cells were cocultured with effector cells at the indicated E: T ratios, and specific lysis was measured after 6 h. Error bars represent mean ± SD. Data are representative of three independent experiments.

**FIGURE 2:**
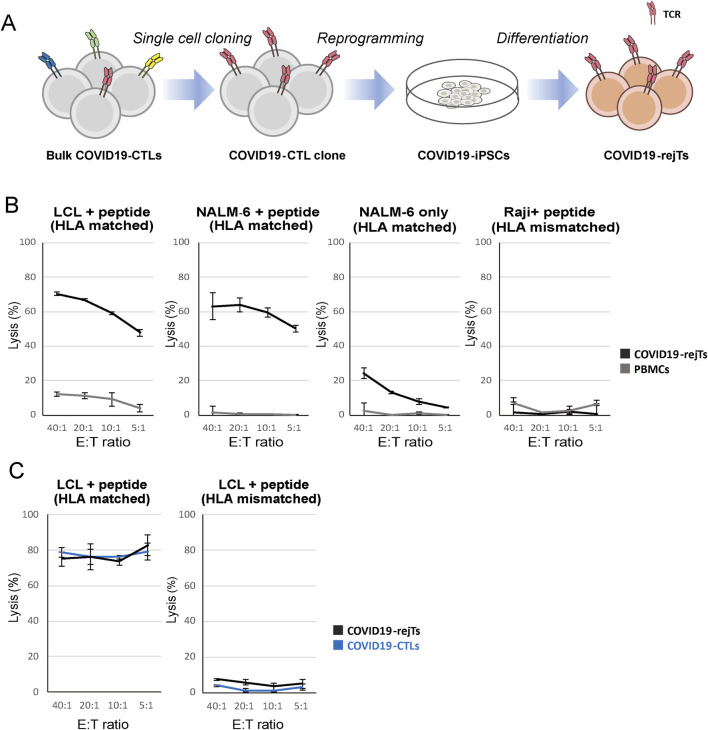
iPSC-derived rejuvenated T cells (COVID19-rejTs) were generated from SARS- CoV-2 spike protein-specific CTL clones and retained antigen-specific, HLA-restricted cytotoxicity. **(A)** Schematic illustrations of generation of COVID19-rejTs. T-iPSCs established from SARS-CoV-2 spike protein-specific CTL clones were differentiated into COVID19-rejTs. **(B)** *In vitro* ^51^Cr release assay of PBMCs and COVID19-rejTs (both HLA-A*02:01-restricted) against LCLs (HLA-A*02:01), NALM-6 (HLA-A*02:01), and Raji (HLA-A*24:02). PBMC controls were derived from the same HLA-A*02:01 donor used to generate the COVID19-CTL clone and COVID19-iPSCs. Target cells were pulsed with S269 peptide prior to co-culture at the indicated E: T ratios, except for NALM-6, which was tested without peptide. Specific lysis was measured after 6 h. Error bars indicate mean ± SD of technical triplicate wells. Data are representative of three independent experiments. **(C)** *In vitro* ^51^Cr release assay of SARS-CoV-2 specific CTLs and COVID19-rejTs (both HLA-A*02:01-restricted) against HLA-A*02:01 and HLA-A*24:02 LCLs, respectively. Target cells were pulsed with S269 peptide prior to co-culture at the indicated E: T ratios. Specific lysis was measured after 6 h. Error bars indicate mean ± SD of technical triplicate wells. Data are representative of three independent experiments.

**FIGURE 3:**
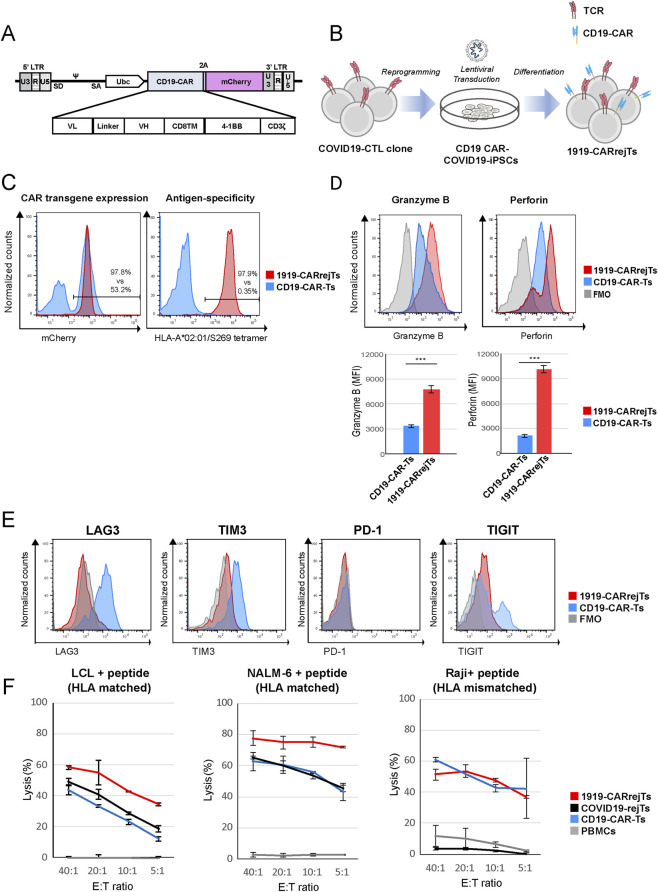
Introduction of a CD19-CAR into COVID19-iPSCs generates dual-antigen recognition 1919-CARrejTs exhibiting more cytotoxicity and less exhaustion compared to conventional CD19-CAR-Ts. **(A)** Schematic representation of CD19-BBz CAR lentiviral vector, containing Ubc promoter. The construct including a CD8 signal peptide, anti-CD19 scFv, CD8a stalk 4-1BB co-stimulatory, and CD3ζ domain. The CAR sequence is linked to the fluorescent reporter mCherry via a self-cleaving 2A-like sequence. **(B)** Schematic illustrations of generation of 1919-CARrejTs. T-iPSCs established from SARS-CoV-2 spike protein-specific CTL clones were transduced with a lentiviral CD19-CAR vector generating CD19-CAR T-iPSCs, which were subsequently differentiated into 1919-CARrejTs. **(C)** Flow cytometric analysis of SARS-CoV-2 antigen specificity and CD19-CAR expression in CD19-CAR-Ts and 1919-CARrejTs. SARS-CoV-2 antigen specificity was detected using a PE-conjugated HLA-A*02:01/S269 tetramer (left). Expression of CD19-CARs was detected by the linked fluorescent reporter mCherry (right). The data shown are representative of at least three independent experiments. **(D)** Flow cytometric analysis of cytotoxic effector molecule expression in CD19-CAR-Ts (blue) and 1919-CARrejTs (red). Histograms of intracellular expression of granzyme B and perforin are shown (upper panel). Bar plots of mean fluorescence intensity (MFI) are shown (lower panel). Error bars indicate mean ± SD. ***p < 0.001 by unpaired Student’s *t*-test. Data are representative of three independent experiments. Background fluorescence comparisons using FMO controls (gray) are shown in Supplementary Figure S1. 1919-CARrejTs were used for background fluorescence-minus-one (FMO) control. **(E)** Expression of exhaustion markers on CD19-CAR-Ts (blue) and 1919-CARrejTs (red). Histograms show expression levels of LAG-3, TIM-3, PD-1, and TIGIT on CD3^+^ cells. The data shown are representative of at least three independent experiments. 1919-CARrejTs were used for background FMO control (gray). **(F)** *In vitro* ^51^Cr release assay of CD19-CAR-Ts, COVID19-rejTs, and 1919-CARrejTs (effectors) against LCLs, NALM-6, and Raji (targets). Target cells were pulsed with S269 peptide prior to co-culture at the indicated E: T ratios. Specific lysis was measured after 6 h. Error bars indicate mean ± SD of technical triplicate wells. Data are representative of three independent experiments.

The cytotoxic activity of 1919-CARrejTs, COVID19-rejTs, CD19-CAR-Ts was evaluated in a standard 6-h ^51^Cr release assay at different effector-to-target (E:T) ratios (40:1, 20:1, 10:1, and 5:1) (Rooney et al., 2014). Target cells used in these assays included LCLs, NALM-6, and Raji cells as specified in each figure (Figures 1–3). Where indicated, targets were pulsed with S269 peptide prior to coculture. Target cells were labeled with sodium chromate (^51^Cr) and cocultured with T cells, with or without S269 loading. After incubation, 50 μL of supernatant was transferred from each well to LumaPlate-96 wells (PerkinElmer, Waltham, MA) and dried overnight. Released ^51^Cr was quantitated using a TopCount microplate scintillation counter (PerkinElmer), with % specific lysis = {([experimental ^51^Cr release] – [spontaneous ^51^Cr release])/([maximal ^51^Cr release] – [spontaneous ^51^Cr release])} × 100. Each condition was assayed in technical triplicate wells per experiment. Unless otherwise stated, ^51^Cr-release assays were repeated in three independent experiments performed on different days using independently prepared effector and target cells.

### 2.10 Flow cytometry

Samples were processed using LSRFortessa and FACSCalibur cytometers (BD Biosciences, San Jose, CA). The results were analyzed using FlowJo software 10.10.0 (Tree Star, Ashland, OR). After cells were incubated with the appropriate concentration of fluorescence-conjugated monoclonal antibody cocktail for 30 min at 4 °C, they were washed with phosphate-buffered saline. Chromophore-conjugated monoclonal antibodies directed against surface antigens were used; they included APC mouse anti-human CD4 antibody (BioLegend, 317416), APC mouse anti-human PD-1 antibody (BioLegend, 329908), APC/cyanine7 mouse anti-human CD3 antibody (BioLegend, 300318), APC/cyanine7 mouse anti-human TIM3 antibody (BioLegend, 345026), fluorescein isothiocyanate (FITC) mouse anti-human CD19 antibody (BioLegend, 302206), PE mouse anti-human TIGIT antibody (BioLegend, 372704), BV421 mouse anti-human LAG3 antibody (BioLegend, 369314), Pacific Blue mouse anti-human CD8 antibody (BioLegend, 301023). CAR expression was evaluated based on mCherry fluorescence in CAR-transduced cells. PE-conjugated HLA-A*02:01/S269 tetramer and HLA-A*24:02/S448 tetramer (MBL) were used to detect antigen-specificity.

### 2.11 Sequential rechallenge assay

**FIGURE 4:**
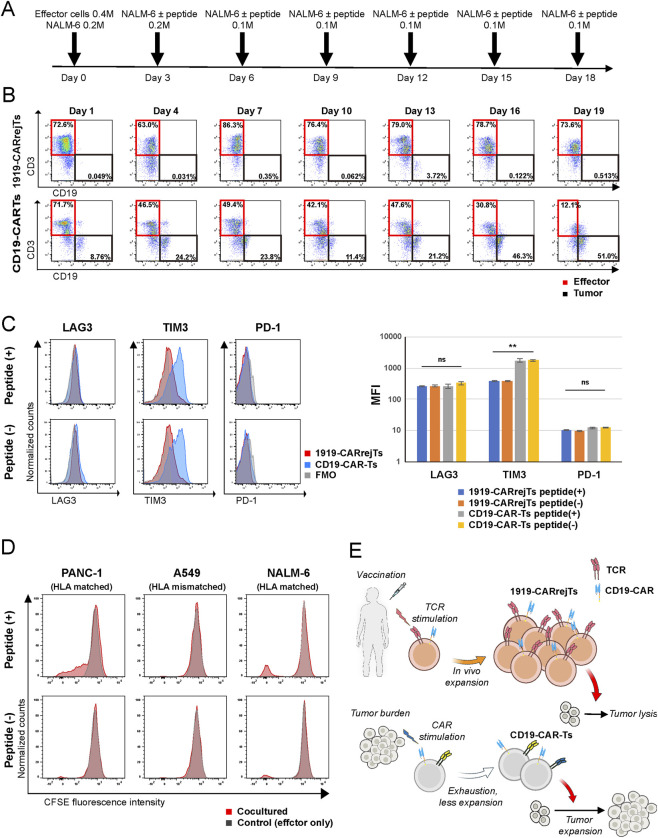
1919-CARrejTs show sustained tumor suppression in a sequential rechallenge model and exhibit enhanced proliferation upon TCR-mediated restimulation, supporting a vaccine-boosting strategy. **(A)** Schematic illustrations of the sequential rechallenge assay. CD19-CAR-Ts and 1919-CARrejTs were initially cocultured with CD19^+^ NALM-6 cells, followed by sequential rechallenges at defined intervals. At each rechallenge, effector cells were re-exposed to target tumor cells under peptide-loaded or non-loaded conditions. Effector-to-target ratios were assessed by flow cytometry using CD19 and CD3 markers. **(B)** Representative flow cytometry plots during sequential rechallenge assay. Tumor burden was quantified at each time point by flow cytometric analysis of CD19^+^ populations. Showing gating of CD19^+^ (tumor, black) and CD3^+^ (effector, red) populations at each rechallenge under peptide-loaded conditions. Single-positive gates are indicated by boxes. **(C)** Expression of exhaustion markers on effector cells at the endpoint of the sequential rechallenge assay. Flow cytometry histograms (left) show the expression of LAG-3, TIM-3, and PD-1 on CD3^+^ effector cells. MFIs are quantified in bar plots (right). Error bars indicate mean ± SD. **p < 0.01 by unpaired Student’s *t*-test. Data are representative of experiments performed in triplicate. 1919-CARrejTs were used for background FMO control (gray). **(D)** CFSE dilution of 1919-CARrejTs following overnight coculture with or without target cells, representing cocultured or control (effector only). Target cells were used with or without S269 peptide loading. Representative histograms show fluorescence intensity of CFSE, indicating the successive cell divisions. Data are representative of three independent experiments. **(E)** Conceptual schematic illustrations of the *in vivo* behavior of 1919-CARrejTs. CAR-only signaling leads to exhaustion and limited persistence in peripheral blood-derived CD19-CAR-Ts, whereas TCR restimulation through vaccination induces sustained proliferation and persistence of 1919-CARrejTs. Image courtesy of the NIH BioArt collection.

CD19-CAR-Ts and 1919 CARrejTs were first cocultured with CD19^+^ NALM-6 tumor cells for an initial challenge and then subjected to sequential rechallenges. The initial coculture was established with 0.4 × 10^6^ effector cells and 0.1 × 10^6^ NALM-6 cells. For each subsequent rechallenge, NALM-6 cells were supplemented at 0.1 × 10^6^ cells. At each rechallenge, effectors were re-exposed to tumor cells under peptide-loaded or non-loaded conditions. Effector and tumor cells were cocultured in NS-A2 medium. Immediately before each rechallenge and at the final time point, tumor to effector ratios were quantified by flow cytometry based on CD19 (tumor) and CD3 (T cells) expression, according to the protocol shown in Figure 4A.

### 2.12 Lymphocyte proliferation assay (CFSE dilution)

Proliferation of 1919-CARrejTs was quantified by carboxyfluorescein succinimidyl ester (CFSE) dilution. Effector cells were labeled with 5 μM CFSE (CellTrace™ CFSE Cell Proliferation Kit, Thermo Fisher Scientific) for 20 min at 37 °C and were then washed twice before coculture. Labeled effectors were cocultured with target cells for 72 h at a 4:1 E:T ratio under the following conditions: (i) PANC-1 ± S269 peptide (HLA-matched, CD19^−^), (ii) A549 ± S269 peptide (HLA-mismatched, CD19^−^), (iii) NALM-6 ± S269 peptide (HLA-matched, CD19^+^), or (iv) a no-tumor control containing effector cells only. After incubation, cells were harvested, stained with anti-CD3 antibody (BioLegend), and analyzed for CFSE dilution of viable CD3^+^ T cells detected by flow cytometry.

### 2.13 Statistical analysis

All data are presented as mean ± SD as stated in the figure legends. Results were analyzed by an unpaired Student’s *t*-test as stated in the text. All statistical analyses were performed using Excel (Microsoft, Redmond, WA) and Prism (GraphPad, San Diego, CA) programs. Values of *p <* 0.05 were considered significant.

## 3 Results

### 3.1 Vaccination induced spike protein-specific CD8^+^ T cells in healthy donors

SARS-CoV-2 spike protein-specific CD8^+^ T cells were not detectable or detectable at low frequencies in PBMCs from healthy donors carrying HLA-A*02:01 or HLA-A*24:02, accounting for 0.000% and 0.015% of total CD3^+^ T cells, respectively. Following SARS-CoV-2 mRNA vaccination, the frequencies within the CD3^+^ lymphocyte gate clearly increased to 0.186% and 0.674%, respectively (Figure 1A). To generate antigen-specific CTLs *in vitro*, PBMCs were obtained from healthy donors more than 1 year after their last vaccination and cocultured with autologous DCs pulsed with the corresponding SARS-CoV-2 spike peptides (S269 for HLA-A*02:01 and S448 for HLA-A*24:02). Tetramer staining confirmed the expansion of SARS-CoV-2 spike-specific CTLs following DC stimulation (0.154% and 0.089% of CD3^+^ T cells for HLA-A*02:01 and HLA-A*24:02, respectively) and subsequently single-cell cloned (83.8% and 96.9%, respectively) (Figure 1B). To examine cytotoxic functions, we performed 6-h ^51^Cr -release assays. SARS-CoV-2 spike-specific CTLs exhibited cytotoxicity against HLA-matched, peptide-loaded LCLs but not against HLA-mismatched LCLs (HLA-A*02:01 restricted CTLs: 78.7% ± 3.0% versus 4.0% ± 0.6%, *p <* 0.001, and HLA-A*24:02 restricted CTLs: 82.8% ± 1.0% versus 3.2% ± 1.3%, *p <* 0.001) at an E:T ratio of 40:1 (Figure 1C). These results confirmed that TCR stimulation by SARS-CoV-2 spike-protein vaccination increased proliferation of antigen-specific CTLs and that the generated CTLs exhibited cytotoxic activity against SARS-CoV-2 spike-peptide pulsed LCLs.

### 3.2 iPSC-derived SARS-CoV-2 spike protein-specific CTLs retained antigen-specific cytotoxicity

SARS-CoV-2 specific CTLs were cloned, reprogrammed into iPSCs, and differentiated into rejuvenated CTLs (COVID-19 rejTs), as outlined in the schematic overview (Figure 2A). In 6-h ^51^Cr -release assays, COVID19-rejTs mediated antigen-specific cytotoxicity. They showed robust killing against HLA-matched, spike peptide loaded LCLs and NALM-6 targets, with specific lysis of 70.4% ± 1.1% and 63.1% ± 7.7%, respectively, at an E:T ratio of 40:1, whereas activated T-cells from HLA-matched healthy donors showed minimal lysis of 12.0% ± 1.2% and 1.6% ± 3.6%, respectively (Figure 2B). When tested against HLA-matched but peptide-unloaded NALM-6 targets, only low levels of unspecific lysis was detected compared to activated T cells (23.4% ± 2.8% versus 1.2% ± 2.6%, at an E:T ratio of 40:1). On the other hand, COVID19-rejTs showed minimal cytotoxicity against HLA-mismatched, spike peptide-loaded Raji cells, with specific lysis comparable to activated T cells (1.4% ± 0.7% versus 6.9% ± 8.5%, at an E:T ratio of 40:1). (Figure 2B).

We next compared the lysis of COVID19-rejTs with that of original COVID19-CTL clone against HLA-matched, peptide-loaded LCLs. COVID19-rejTs demonstrated antigen-specific cytotoxicity comparable to that of the original CTL clones against HLA-matched, peptide-loaded LCLs (75.1% ± 4.0% versus 78.7% ± 3.0%, at an E:T ratio of 40:1), whereas neither effector cells mediated detectable lysis against HLA-mismatched LCLs, even when peptide-pulsed (7.4% ± 0.3% versus 4.0% ± 0.6%) (Figure 2C). These results confirm that cytotoxicity of COVID19-rejTs is retained in an HLA-restricted antigen-specific manner, even after iPSC reprogramming and redifferentiation.

### 3.3 Generation of dual-antigen recognition 1919-CARrejTs by introduction of CD19-CAR into COVID19-iPSCs

To generate dual-antigen recognition rejTs, CD19-CAR-2A-mCherry (Figure 3A) was introduced into COVID19-iPSCs originated from HLA-A*02:01-restricted COVID19-CTLs, then differentiated into T cells, as outlined in the schematic overview (Figure 3B). The resulting product, referred to as 1919-CARrejTs, represent CD19-CAR-expressing rejTs that retain spike protein-specific TCR. Next, we evaluated CD19-CAR transgene expression and spike protein specificity in 1919-CARrejTs by flow cytometry based on mCherry detection and HLA-A*02:01/S269 tetramer staining. Both CAR transgene expression (mCherry positivity) and tetramer positivity were nearly 100%, confirming that 1919-CARrejTs uniformly expressed CD19-CAR and maintained spike protein-antigen specificity (Figure 3C). In parallel, we generated conventional CD19-CAR-T cells from peripheral blood using the same CAR construct. In these cells, CAR expression was detected in 53.2% of CD3^+^ T cells, reflecting transduction efficiency, whereas spike protein-specific TCRs were nearly undetectable (0.35%) (Figure 3C).

### 3.4 1919-CARrejTs exhibit increased cytotoxicity and decreased exhaustion compared to conventional CD19-CAR-T cells

To compare T-cell functions of 1919-CARrejTs with that of conventional CD19-CAR-T cells, expression levels of cytotoxic molecules and exhaustion markers were evaluated by flow cytometric analysis. 1919-CARrejTs showed higher expression levels of intracellular cytotoxic molecules (granzyme B and perforin) than conventional CD19-CAR-T cells, with mean MFI values of 7771.7 ± 446.6 versus 3330.3 ± 211.2 (*p <* 0.001), and 10162 ± 1273 versus 2129.3 ± 241.4 (*p <* 0.001), respectively (Figure 3D). Furthermore, expression levels of T-cell exhaustion markers LAG-3, TIM-3, and TIGIT, were decreased in 1919-CARrejTs compared with conventional CD19-CAR-T cells (Figure 3E). These results demonstrate that 1919-CARrejTs exhibited more cytotoxic activity and less exhaustion compared to conventional CD19-CAR-T cells.

### 3.5 Dual-antigen recognition improves immediate effector function under antigen presentation

Six-hour ^51^Cr -release assays revealed that 1919-CARrejTs exhibit stronger cytotoxicity against LCL targets (CD19^+^, HLA-matched, spike peptide-loaded) compared to conventional CD19-CAR-T cells, with specific lysis of 58.5% ± 1.2% versus 43.7% ± 3.3% (*p =*0.018) at an E:T ratio of 40:1, respectively (Figure 3F, left). Similar results were observed when comparing 1919-CARrejTs and conventional CD19-CAR-T using NALM-6 (CD19^+^, HLA-matched, spike peptide-loaded), exhibiting specific lysis of 77.5% ± 4.6% versus 62.5% ± 5.6% (*p =*0.023) at an E:T ratio of 40:1, respectively (Figure 3F, middle). When Raji cells (CD19^+^, HLA-mismatched, spike peptide-loaded) were used, the difference in cytotoxicity was lost, with specific lysis of 53.5% ± 4.1% versus 51.4% ± 1.9% (*p =*0.478) at an E:T ratio of 20:1, respectively (Figure 3F, right). These results are consistent with our previous findings that dual-antigen recognition amplifies immediate cytotoxicity when the targets have antigens recognizable by both CAR and TCR (Harada et al., 2022).

### 3.6 1919-CARrejTs exhibit sustained tumor suppression throughout serial tumor rechallenges

To assess cytotoxicity of 1919-CARrejTs under repeated tumor exposure, we utilized a sequential rechallenge model in which effectors were cocultured with NALM-6 and then re-exposed at prespecified intervals. Four arms were tested under identical schedules: CD19-CAR-T cells ± spike protein peptide and 1919-CARrejTs ± spike protein peptide. E:T ratios were measured by flow cytometry (CD3 for effector cells; CD19 for target tumor cells (Figure 4B; Supplementary Figure S3). In this setting, conventional CD19-CAR-T cells showed progressive tumor outgrowth upon repeated tumor exposure. Tumor burden increased from 8.76% at day 1%–51.0% by day 19 (end of the seventh challenge). In contrast, 1919-CARrejTs showed sustained tumor suppression, with tumor fractions remaining below 5% throughout all rechallenge cycles. End-of-experiment analysis within the CD3^+^ gate showed lower TIM-3 expression in 1919-CARrejTs than in conventional CD19-CAR-T cells with TCR stimulation, with mean MFI values of 388.3 ± 12.0 versus 1723.8 ± 195.3 (*p <* 0.001), respectively. Similar results were observed also in the absence of TCR stimulation, with mean MFI values of 384.5 ± 6.5 versus 1762.2 ± 87.0 (*p <* 0.001). In contrast, expression levels of PD-1 and LAG-3 were similar across all arms (Figure 4C). In summary, our data demonstrate that 1919-CARrejTs not only exhibited stronger cytotoxic activity than conventional CD19-CAR-T cells, but also maintain effective tumor suppression even after multiple rounds of tumor rechallenge. Also, 1919-CARrejTs eradicated tumor cells independently of TCR stimulation with spike protein peptide.

### 3.7 TCR stimulation exhibits increased proliferation compared to CAR stimulation

We performed CFSE dilution assays of 1919 CARrejTs under three coculture conditions to distinguish the effects of TCR- and CAR-mediated cues on proliferation: (i) PANC-1 cells ± spike protein peptide (HLA-matched, CD19^−^; TCR-dependent), (ii) A549 cells ± spike protein peptide (HLA-mismatched, CD19^−^; negative control), and (iii) NALM-6 cells (CD19^+^; CAR-only stimulation) (Figure 4D). 1919-CARrejTs showed efficient proliferation when cocultured with spike protein peptide-loaded PANC-1, while proliferation levels were low when cocultured with spike protein peptide-unloaded PANC-1. As expected, 1919 CARrejTs with A549 cells induced low proliferation levels, regardless of spike protein peptide presence. Finally, 1919-CARrejTs showed limited proliferation upon CAR stimulation alone, whereas peptide loading of NALM-6 induced additional proliferation, consistent with an additive contribution of TCR signaling under concurrent CAR stimulation. These results suggest that native TCR stimulation augments CAR-driven proliferative cues. Together with the results from the sequential rechallenge model, these findings suggest that CARrejTs maintain cytotoxic activity over longer periods of time compared to conventional CAR-T cells, with TCR signaling serving as an auxiliary cue that supports their expansion and persistence.

## 4 Discussion

Major obstacles that limit the efficacy of CAR-T cell therapy include suppression of T-cell function by the tumor microenvironment and CAR-T cell exhaustion. To overcome these barriers, various next-generation CAR-T cells have been developed, such as cytokine secreting “armored” CAR-T cells (Hawkins et al., 2021), multiantigen-targeting CAR designs (Kloss et al., 2013), immune checkpoint-disrupted CAR-T cells (Rupp et al., 2017), allogeneic CAR-T cells (Qasim et al., 2017), and iPSC-derived functionally rejuvenated T-cells (Nishimura et al., 2013). These approaches have shown efficient antitumor activity in preclinical models or early clinical trials.

In addition, the concept of “vaccine synergy” has been developed to leverage native TCR signaling for restimulation of CAR-T cells, thereby enhancing their antitumor activity and *in vivo* persistence. Previous reports using varicella zoster virus vaccines (Tanaka et al., 2017) and cytomegalovirus peptide vaccines (Wang et al., 2015) that activate virus-specific native TCRs in CAR-T cells have suggested that vaccine-induced TCR restimulation is expected to promote CAR-T cell expansion and enhance anti-tumor responses in preclinical models. In this setting, SARS-CoV-2 mRNA vaccines can be particularly attractive for “vaccine synergy” with CAR-T cells, because they are repeatedly administered worldwide. In addition, unlike the general population, patients with hematological malignancies are still at high risk of progressing to life-threatening severe COVID-19 infections, even in the Omicron era. Furthermore, a significant number of immunocompromised patients undergo an abnormally prolonged disease course known as “persistent COVID-19”, in which the SARS-CoV-2 infection continues for weeks to months, and thereby postponing and terminating scheduled chemotherapy courses (Herishanu et al., 2021; Zerbit et al., 2022; Yasuda et al., 2025). Therefore, patients with hematological malignancies are particularly recommended to receive repeated COVID-19 vaccinations (Luque-Paz et al., 2023; Pothast et al., 2025). Furthermore, it has been reported that SARS-CoV-2 specific CTLs can be rapidly expanded *ex vivo* for treatment of COVID-19 in hematopoietic stem cell transplant recipients (Keller et al., 2020). Therefore, SARS-CoV-2 mRNA vaccine as a vaccine synergy antigen with SARS-CoV-2 spike protein specific T-cells may not only be effective towards B-cell malignancies, but also may provide protection against COVID-19.

We previously developed a dual-antigen recognition rejT platform to address the problem of CAR-T cell exhaustion. In this platform, EBV LMP2-specific CTLs are reprogrammed into iPSCs, a CD19-CAR is introduced, and the cells are differentiated into dual-antigen recognition LMP2-specific CARrejTs that respond through both their native TCR and the CAR (Harada et al., 2022). Analysis showed that CD19-CARrejTs were enriched with central memory T-cells and exhibited stronger cytotoxicity compared to single-antigen targeted rejTs against EBV- and CD19-positive lymphoma cells. The 1919-CARrejTs expressing both CD19-CAR and spike protein-specific TCRs introduced in this report were generated utilizing the technology from LMP2-specific CARrejTs. In the present work, we extended this platform to SARS-CoV-2 spike-specific T-cells and establish proof of concept for vaccine-induced enhancement of CARrejTs. We focused on clarifying how vaccine-induced TCR restimulation contributes to proliferation and cytotoxicity of 1919-CARrejTs. To examine this, we used a sequential rechallenge assay to model repeated tumor exposure and a CFSE based proliferation assay to directly compare, within the same cells, the effects of addition of TCR mediated stimulation on CAR-T cell proliferation.

Our observations that 1919-CARrejTs expressed higher levels of granzyme B and perforin and lower levels of LAG-3, TIM-3, and TIGIT than conventional CD19-CAR-T cells (Figure 3E) support the idea that iPSC mediated rejuvenation can partially reset exhaustion associated transcriptional and epigenetic changes. The sustained tumor suppression achieved by 1919-CARrejTs in the sequential rechallenge assay further suggests that rejTs are more resistant to repeated antigen exposure, consistent with previous findings using EBV or human papillomavirus (HPV)- specific rejTs (Ando et al., 2020; Honda et al., 2020; Furukawa et al., 2023). Our data also support the idea that stimulation through the native TCR and through the CAR are not functionally equivalent and may influence T-cell fate in different ways. In the CFSE assay, HLA-matched, spike protein peptide-loaded CD19^−^ targets, which provide antigen-specific, HLA-restricted TCR engagement, induced strong proliferation of 1919-CARrejTs, whereas CAR stimulation alone by CD19^+^ NALM-6 cells led to only modest proliferation. These findings are consistent with previous observations that virus specific T-cells can maintain responsiveness through their native TCR, and that TCR restimulation can partially restore CAR dependent antitumor activity (Tanaka et al., 2017; Wachsmann et al., 2022). Together with the sequential rechallenge data, these results suggest that TCR-mediated signals could provide a complementary advantage for robust proliferation and long-term function of dual-antigen recognition CARrejTs.

Activation of the native TCR engages a broad signaling network. In contrast, CAR signaling is initiated through CD3ζ ITAMs together with the engineered costimulatory domains in the CAR architecture, and exhibits a more skewed and restricted proximal signaling manner compared to that of native TCR (Huang et al., 2023). These differences in proximal signaling may have contributed to the stronger proliferative response we observed after TCR-mediated stimulation compared with CAR-only stimulation in 1919-CARrejTs. Direct analysis of the downstream molecular mechanisms, including transcriptomic analysis, were beyond the scope of this study but will be important as future work. Our findings support the concept of “vaccine-synergy” in which mRNA vaccine-induced TCR restimulation in an HLA restricted manner can be utilized to promote proliferation and persistence of dual-antigen recognition rejTs. Also in this model, we found that CAR signaling functions mainly as a tumor targeting module, and contribution to rejT proliferation and persistence was limited. (Figure 4E).

The rejT platform provides a scalable source of antigen specific CTLs and thereby enables the development of “off-the-shelf” CARrejTs. CARrejTs could be manufactured in advance targeting frequently found HLA subgroups, administered on demand, and later boosted *in vivo* using vaccines (Ando and Nakauchi, 2017; Iriguchi et al., 2021). We have already started investigator-initiated phase-1 clinical trials of EBV- and HPV- specific rejTs for EBV associated lymphomas (JRCT2033210485) and cervical cancer (jRCT2033240591), respectively, to evaluate the safety and feasibility of this approach. Therefore, we conclude that combining CARs with a vaccine booster strategy would represent a next step in the evolution of this platform. Future studies using mouse tumor models will be important to establish *in vivo* proof-of-concept for vaccine-driven boosting of dual-antigen recognition CARrejTs, as demonstrated previously in related preclinical evaluations of iPSC-derived dual-target CARrejTs (Harada et al., 2022).

## Data Availability

The raw data supporting the conclusions of this article will be made available by the authors, without undue reservation.
