# Supplementary figures and images for: Dual-antigen recognition iPSC-derived CAR-T cells for B-cell malignancies: establishment of a COVID-19 vaccine synergy strategy
Source: Front Cell Dev Biol. 2026 Feb 26;14:1772146. doi: 10.3389/fcell.2026.1772146 (PMC12979464; doi:10.3389/fcell.2026.1772146)

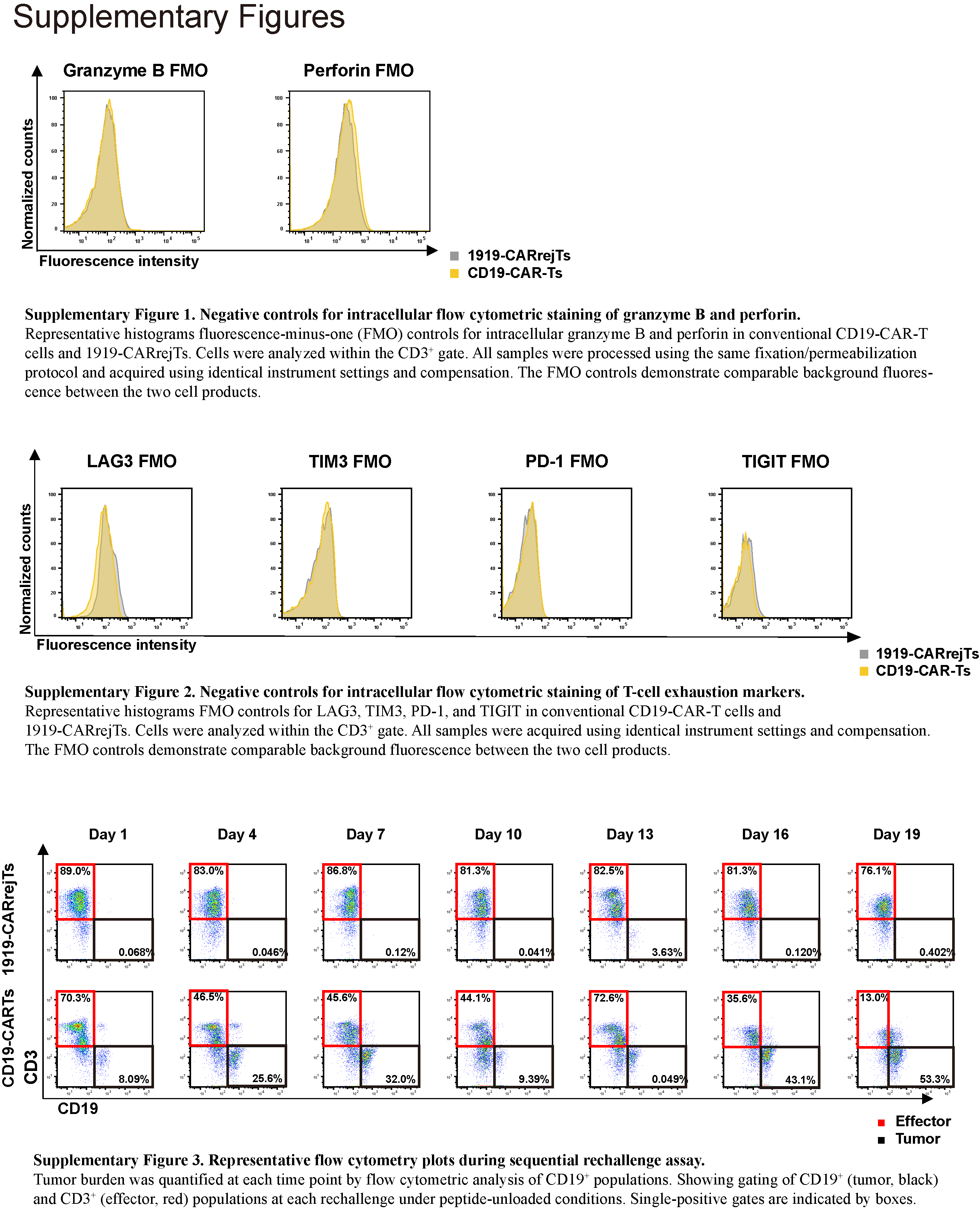

Supplement: Supplementary file 1 [file Image1.tif]
